# Effects of Direct Instruction and Strategy Modeling on Upper-Primary Students’ Writing Development

**DOI:** 10.3389/fpsyg.2017.01054

**Published:** 2017-06-30

**Authors:** Paula López, Mark Torrance, Gert Rijlaarsdam, Raquel Fidalgo

**Affiliations:** ^1^Department of Psychology, Sociology and Philosophy, Faculty of Education, University of LeónLeón, Spain; ^2^Division of Psychology, Nottingham Trent UniversityNottingham, United Kingdom; ^3^Research Institute for Child Development and Education, University of AmsterdamAmsterdam, Netherlands

**Keywords:** writing, strategy-focused instruction, components analysis, modeling, direct instruction

## Abstract

Strategy-focused instruction is one of the most effective approaches to improve writing skills. It aims to teach developing writers strategies that give them executive control over their writing processes. Programs under this kind of instruction tend to have multiple components that include direct instruction, modeling and scaffolded practice. This multi-component nature has two drawbacks: it makes implementation challenging due to the amount of time and training required to perform each stage, and it is difficult to determine the underlying mechanisms that contribute to its effectiveness. To unpack why strategy-focused instruction is effective, we explored the specific effects of two key components: direct teaching of writing strategies and modeling of strategy use. Six classes (133 students) of upper-primary education were randomly assigned to one of the two experimental conditions, in which students received instruction aimed at developing effective strategies for planning and drafting, or control group with no strategy instruction: Direct Instruction (*N* = 46), Modeling (*N* = 45), and Control (*N* = 42). Writing performance was assessed before the intervention and immediately after the intervention with two tasks, one collaborative and the other one individual to explore whether differential effects resulted from students writing alone or in pairs. Writing performance was assessed through reader-based and text-based measures of text quality. Results at post-test showed similar improvement in both intervention conditions, relatively to controls, in all measures and in both the collaborative and the individual task. No statistically significant differences were observed between experimental conditions. These findings suggest that both components, direct teaching and modeling, are equally effective in improving writing skills in upper primary students, and these effects are present even after a short training.

## Introduction

Theories of the psychological processes underlying how people write extended text – the processes by which, for example, students write essays and researchers write papers – have historically had two main strands. Writing is characterized as a problem solving process, in which the writer makes deliberate and explicit decisions about content, structure, rhetoric, and word choice (Flower and Hayes, 1980; Bereiter and Scardamalia, 1987; Hayes, 1996). Writing is also cognitively demanding: the processes associated with text production must be coordinated within the constraints imposed by a limited capacity of working memory (Kellogg, 1988, 1999; McCutchen, 1996; Torrance and Galbraith, 2006). Therefore writers must coordinate several cognitively costly activities including retrieval of prior knowledge, planning and structuring content, formulating sentences, and monitoring output. At the same time, writers need to maintain in mind their communicative goals and the needs of their audience (Flower and Hayes, 1980; Fayol, 1999). Writing is particularly demanding task for young writers. Writers who have not yet fully developed low-level transcription skills – who are not yet able to plan fluently and accurately and execute sentences that are grammatically correct and words that are accurately spelled and neatly written – face a combined challenge. They struggle to produce accurate sentences, and the consequent additional effort draws resources away from the higher-level problem solving activities necessary to generate well-structured and content-rich text. Arguably therefore, as Graham and Harris (2000) observe, writing competence requires not only automatization of transcriptions skills but also self-regulation in order to handle high-level cognitive processes of writing such as planning and revision, which are directly related to the production of high-quality texts (Limpo et al., 2014; for a review see Berninger, 2012).

Strategy-focused writing instruction aims to teach developing writers strategies that give them executive (self-regulatory) control over their own writing processes. Several meta-analyses (Graham and Perin, 2007; Graham et al., 2012; Graham and Harris, 2014) have indicated that strategy-focused instruction is the most effective approach to improve students writing, relative to the other types on instruction identified in their meta-analyses, with typically large positive effects on the quality of students’ texts. This approach aims to give students explicit strategies for regulating both what they write and the processes that they adopt when writing it (Alexander et al., 1998; Harris et al., 2008).

Programs of strategy-focused instruction tend to have multiple components, and these vary to some extent across different implementations (Pressley and Harris, 2006; Harris et al., 2011). However, instruction typically includes activities aimed at activating relevant prior knowledge, direct instruction aimed at giving declarative meta-knowledge about appropriate writing strategies, typically based around various mnemonics, modeling of writing strategies in which the instructor “thinks aloud” in front of learners demonstrating a strategy while composing, and scaffolded practice. Merrill (2002) refers to these five components at the “First Principles” of instruction. The aim is for a progressive decrease in scaffolding, with strategies moving from being something that the teacher tells the students to do, to internalized self-talk by which the student regulates their own writing behavior (Pressley and Harris, 2006; Graham and Harris, 2014).

As we have noted, a number of evaluations of instructional programs based on these components have found that the programs as a whole are successful, and more successful than other approaches to writing instruction. However, these studies necessarily have evaluated a package of instructional components. It is unclear whether all or just some of those components contribute to the positive outcome. Therefore several researchers have pointed to the need for component analyses (Graham and Harris, 1989; De La Paz, 2007; Brunstein and Glaser, 2011). Such studies are critical for both theoretical and educational reasons. From a theoretical perspective, understanding the relative contribution of the different components of strategy-focused instruction gives insight into the underlying mechanisms of writing development (Sawyer et al., 1992). Understanding the relative efficacy of different instructional components in a “package intervention” also contributes to understanding of students’ learning processes (Hopwood, 2007). From an applied perspective, full strategy-focused interventions typically do not fit well within the normal school curriculum, and teachers are liable to selectively include some but not all components in their classroom practice (De La Paz, 2007). This is for several reasons. Implementing strategy-focused instruction can be challenging for teachers. Some components, and particularly modeling, will often be outside of the teacher’s skills set and are typically, in the US at least, not well-supported in professional development (Harris et al., 2009). Also, the best-known approach to strategy-focused instruction (Self-Regulation Strategy Development; e.g., Graham et al., 2000; Harris et al., 2006) requires teaching individual or small groups of students following a criterion-based approach. The number of instructional sessions devoted to master different components and learning-goals therefore varies across implementation and across students. Adopting this approach in a normal, full-range classroom is typically problematic.

The challenge, therefore, is to identify which of the various components that comprise the strategy-focused approach are necessary to result in substantial positive effects on writing quality when taught to full-range classes. A handful of studies have aimed to compare the efficacy of different components. Several of these have focused on the role of instruction targeted specifically at student motivation, on the role of feedback, and on the effects of peer support (see De La Paz, 2007 for a review). Fewer studies have attempted to explore the specific contribution of the main instructional components detailed above (but see Sawyer et al., 1992; Fidalgo et al., 2011, 2015; Torrance et al., 2015).

Our present focus is on the contribution of direct instruction and of modeling to successful learning. Sawyer et al. (1992) assigned fifth and sixth grade students with learning difficulties to four conditions (1) full strategy-focused instruction, (2) strategy-focused instruction without goal setting and self-monitoring, (3) direct teaching only, and (4) practice control. In the direct instruction condition, the authors removed modeling and collaborative practice, and also instruction on the use of self-talk. The results did not show significant differences between conditions concerning text quality at any measurement occasion at either post-test or delayed post-test. This suggests that direct instruction without modeling is sufficient to improve writing quality, at least in struggling writers. Nevertheless, these results need to be treated with caution, given that the efficacy of modeling seems to be heavily dependent on several factors. For example, in line with Braaksma et al. (2002) findings, weak students benefit more when they can observe weak models. As the specific sample on Sawyer et al. (1992) presented learning disabilities, it might be the case that students did not benefit from a model that provides them with the opportunity to learn by observation.

The opposite result has also been found. Fidalgo et al. (2011), explored whether strategy-focused instruction remained effective when direct teaching was removed from the program. They compared two seven-session programs, both implemented in full-range classes. In one condition students received full strategy-focused instruction, comprising direct teaching (one session), modeling (two sessions), collaborative (two sessions), and independent practice (two sessions). In the other experimental condition, the direct teaching component was omitted. The results showed that both experimental conditions outperformed the control group in text quality, with no significant differences between conditions. In another study, Fidalgo et al. (2015) analyzed the cumulative contribution of modeling, direct instruction, and collaborative and individual practice. Three sixth-grader classes participated in a lagged-group and cross-panel evaluation. Groups showed significant and substantial gains in text quality after an initial component, taught over two sessions, in which the teacher modeled effective use of specific writing strategies, and students then reflected on what they had observed. These sessions did not include any direct instruction or explicit strategy labeling. Subsequent components gave no significant additional benefit. This finding was observed for both compare-contrast essays and opinion essays. These results suggest that observation of a mastery model followed by a whole-class reflection is sufficient to improve sixth grade students’ writing skills. Nevertheless, this finding should be interpreted cautiously. For example, it might be that *the first blow is half the battle*: the study does not rule out the possibility that starting with Direct Instruction would have resulted in the same effect, and indeed this is what might be predicted based on the finding detailed above. Therefore, a direct comparison of the benefits of these two forms of instruction is needed.

Our goal in the present study, therefore, was to directly compare the contribution of Direct Instruction and Modeling to writing development, through interventions aimed at improving text quality by teaching planning and drafting strategies. For that purpose, we designed two experimental interventions. In the Direct Instruction condition students received explicit declarative knowledge of planning and drafting strategies, supported by mnemonics. In the Modeling condition students were provided with procedural knowledge of how to implement planning and drafting strategies by observing a model. These two experimental conditions were contrasted with a control condition, in which students were taught about the linguistic and discourse features of good text, but were not taught writing process strategies.

Effects of each condition were tested with two tasks, one collaborative and one individual. Several studies have shown positive effects of collaboration on task performance, finding higher quality texts from collaborative writing than from individual writing (Yarrow and Topping, 2001; Wigglesworth and Storch, 2009). As Ohta (2001) pointed out, no two learners have the same strengths and weaknesses, so when working together they can provide scaffolded assistance to each other and achieve a higher level of performance than they may have achieved on their own. Therefore in the present study we wanted to explore whether differential effects resulted from students working alone or observing and commenting on each other’s task, with the aim of encouraging each other to adopt the strategies that they had been taught.

## Materials and Methods

### Design

Six existing classes of 5th and 6th students were randomly assigned to one of two experimental conditions and a control condition, with one 5th and one 6th grade class in each condition. Instruction in all conditions was aimed at training students to produce good quality argumentative texts.

Both experimental conditions received strategy instruction focused on the acquisition of planning and drafting writing strategies. In the *Direct Instruction* condition, the students received direct instruction aimed at delivering declarative knowledge about planning and drafting strategies, supported by the use of mnemonics and graphic organizers. In the *Modeling* condition, students observed an expert model, with the aim of delivering procedural knowledge about the same strategies, but without labeling these strategies or making them explicit. Students in the control condition were taught about the features of good argumentative text, but without any mention to specific strategies for regulating the processes by which these texts might be produced.

Writing performance was assessed before the intervention (pre-test) and immediately after the intervention (post-test). At each measurement occasion students completed two tasks: an individual task and a collaborative writing task performed in pairs that reflected the collaborative learning tasks students practiced during the intervention. All assessment tasks involved writing argumentative texts.

### Participants

The sample comprised 133 Spanish upper-primary students in three 5th grade classes (*N* = 72) and three 6th (*N* = 61) classes. These were all drawn from the same *colegio concertado* (mixed state- and privately-funded) school. Students’ ages ranged from 10 to 12 years (Direct instruction: *M* = 10.48; *SD* = 0.50; Modeling: *M* = 10.75; *SD* = 0.61; Control: *M* = 10.62; *SD* = 0.57), with 50% of female students in direct instruction condition, 46% in the modeling condition and 49% in the control group. Most students came from families with medium to high incomes. An additional 13 students who had existing diagnoses of special educational needs received the same instruction as their peers, but we did not include their data in the analysis.

Prior to intervention, all students received similar writing instruction following a pattern typical in Spanish primary schools. This focuses on the features of different textual genres, and on grammatical and spelling accuracy, and did not include any explicit strategy instruction.

Students were allocated to pairs for the collaborative writing task by the teacher, with children of broadly similar ability within each pair. Students were assigned to roles – either *Writer* or *Helper* – which they maintained throughout the intervention. The teacher also decided which student in a pair was more extrovert, that is which student was more likely to think aloud during the composing task. That student then was selected as the writer, while the other student in the pair was the helper.

### Instructional Programs

The intervention was delivered by one instructor to whole classes, with the same instructor in all cases. All sessions lasted for approximately 55 min in all conditions and followed the same pattern, consisting of two parts. The first 35–40 min of the session involved delivery of the specific instructional content of that session, varying according to condition. In the second part students practiced what they had been taught or had observed, completing a short writing task in pairs. Students with the writer role performed the writing task, verbalizing all their actions and thoughts throughout. Helpers sat next to the writer and monitored their writing processes and output. On the basis of the instruction that they had received in the first part of the session helpers commented on the Writers text, thoughts and, perhaps, processes, identifying issues and suggesting ways in which these might be resolved.

The similarities and differences of the three conditions are summarized in **Table 1**.

**Table 1:** Summary of differences among conditions.

	Direct Instruction	Modeling	Control
**Instructional approach**			
Activation of prior knowledge	+	+	+
Motivation supporting	+	+	+
Practice by pairs	+	+	+
Direct teaching of cognitive writing strategies through mnemonics	+		
Modeling of the use of cognitive writing strategies through thinking aloud		+	
Analysis of high-quality argumentative texts			+
**Instructional content**			
High quality argumentative texts	+	+	+
Planning and drafting writing strategies	+	+	
**Kind of knowledge provided**			
Self-regulated approach	+	+	
Declarative knowledge	+		+
Procedural knowledge		+	

#### Direct Instruction

Teaching of planning (first session) and drafting (second session) was supported by graphic organizers and mnemonics specifically designed for this study. Students were taught the mnemonic “TARE” to scaffold planning their argumentative texts. *Tesis* (*Thesis*) prompted students to identify their stance on the topic (for or against); *Audiencia* (*Audience*) prompted students to think through the specific informational needs of their reader, and the rhetorical strategies that were likely to be most effective in persuading their readers of their position. *Razones* (*Reasons*) prompted students to identify several claims to justify their position. *Ejemplos* (*Examples*) reminded students of the need to evidence these claims.

In the second session students were taught a strategy for drafting their text based around “IDC,” which encouraged planning of specific components of the text: an *Introducción* (*Introduction*) which should interest the reader and clearly state the student’s thesis; *Desarrollo* (*Development*), representing the middle paragraphs in their text in which students were instructed to give reasons and evidence examples in coherence and well-structured manner; and a *Conclusión* (*Conclusion*). Both strategies were supported by graphic organizers that showed the TARE and IDC structure, with explanations and examples.

During collaborative practice, the student with the *Helper* role was asked to support their partner’s (the *Writer’s*) use of the strategy taught in that session, commenting on the Writer’s think aloud with specific reference to the associated mnemonic.

#### Modeling

The instructor started these sessions by explaining that they were about to observe a very good writer planning (first session) or drafting (second session) an argumentative text. Students were asked to give close attention to the model because afterward they would be asked to emulate what they had observed. Modeling involved semi-scripted “think aloud” demonstrating a self-regulating approach to writing argumentative text. The model externalized the internal self-talk that is associated with self-regulated strategy use, while implementing the same self-regulated writing procedure that was the intended learning outcome of the Direct Instruction intervention. The instructor therefore articulated her stance on the topic, setting reader-focused goals, generating supporting ideas and so forth as she produced her written plan (Session 1) and draft of her text (Session 2). Importantly her think-aloud did not make direct reference to strategies and, particularly, did not mention the mnemonics taught in the direct instruction condition. In addition, the instructor included self-talk demonstrating self-belief (“I can do it correctly”; “I am sure that I will get a high mark”) and self-encouragement to remain motivated and attentive (“It is boring, but it is worth the effort”). After modeling was complete students were given a copy of the written output of the modeled writing session – a written plan in Session 1 and a draft essay in Session 2. Finally, students practiced in pairs, with the Writer aiming to emulate what they had observed and the Helper prompting them (e.g., “You are writing down evidence, but I think the teacher stated her own position first”).

#### Control

In both sessions students received examples of high quality argumentative texts about the same topic, with the text in Session 1 arguing one position, and the text is Session 2 arguing the opposite position. The text was read to the class and then students read it individually and answered questions about specific features of structure and content (e.g., “What kind of text you just read?,” “What is the main topic of the text?,” “What evidence do they use?,” “Give at least one argument not mentioned in the text.”). The instructor then led a whole-class discussion about the text, bringing out ideas about the features that made it a successful argument. As in the other two conditions collaborative practice involved creating a written outline (Session 1) and drafting full text (Session 2). In Session 1 Helpers were encouraged to help their partners to generate ideas. In Session 2 they reminded their partners about specific features of high quality argumentative texts, and were also free to contribute additional ideas during the writing task.

#### Implementation and Fidelity

Intervention sessions were 1 week apart and occurred toward the start of the Spring school term. Sessions took place in literacy lessons and they were delivered in all cases by the first author who has previous training and experience in delivering similar interventions. To ensure full implementation of the instructional conditions the program for each session were prescribed in detail. All texts written during the intervention were collected in individual portfolios which enabled us to verify that all students completed all tasks. In addition, all sessions were audio-recorded.

The following procedure ensured that ethical standards were maintained. Parents were informed of research aims via letters in which they gave written informed consent. They were given the opportunity to express concerns and to request that their children’s data not be included in the study. The intervention took place in a common classroom context through several sessions spread in the normal school timetable. Teaching in all conditions covered, and went beyond, the requirements of the school curriculum. After finishing the study, the school was informed about the results of the different instructional conditions, and a specific strategy-focused instruction program and supportive materials, combining elements of the experimental conditions was provided to the students’ normal literacy teacher to be implemented with the control group students.

### Instruments and Measures

#### Writing Assessment Tasks

To avoid a contamination of topic and measurement effects, writing performance was assessed by students writing argumentative essays with topics counterbalanced across assessment tasks and pre-test and post-test. Topics related to animal captivity and the value of reading (for the collaborative writing tasks) and whether or not sport is a good thing and the value of learning languages (for the individual writing tasks). These were presented on small cards which included specific topic with two pictures and the question “for or against?” For both the collaborative and the individual task, students were provided with two work sheets, one for planning or rough drafting, and one for their final text. Students were told that use of the first work sheet was optional. Students were asked to produce the best essay that they could write. For the collaborative task, the instructor also reminded student’s roles as well as stressed the need to work together on the text. In both assessment tasks, students had 1 hour to write their texts, despite this, none wrote more than 35–40 min.

Texts from both the individual and collaborative assessment tasks were rated holistically through *reader-based measures* and analyzed in detail to generate *text-based measures*.

*Reader-based* measures involved assessing the structure, coherence and overall quality of the texts, using methods adapted from Spencer and Fitzgerald (1993). *Structure* was rated on a four-point scale, with 1 = lack of any obvious structure and 4 = well structured. Raters made decisions based on the extent to which the text had a global framework that made clear the argumentative function of each section of text. *Coherence* was also assessed on a four-point scale, with 1 = incoherent and 4 = entirely coherent. This score was based on whether it was possible to identify the main argument, whether the text presented clear progression of ideas without digressions, whether the student defined a general context, and whether the text maintained local cohesion (sentences followed from each other). *Overall Quality* was assessed on a six-point scale, with 1 = not suitable, hard to understand and 6 = excellent. Scores were based on the extent to which the text included rich ideas, diverse and appropriate, vocabulary, interesting detail, and correct sentence structure, punctuation, and spelling.

Two raters with previous experience of using these measures rated all of the texts independent in three separate rounds, one round per dimension. The inter-rater reliability (Pearson’s *r*) average across assessment moments was high (Individual task: structure, 0.83; coherence 0.92; overall quality, 0.90; Collaborative task: structure, 0.80; coherence, 0.87; overall quality, 0.94).

*Text-based measures* focused on the presence of relatively sophisticated coherence devices within the text. Four types of complex devices were identified: structural ties (e.g., first, *secondly, finally*…), reformulation ties (e.g., *in conclusion…, in other words…, that is to say*…), argumentative ties (e.g., *for example…, therefore…, however*…), and meta-structural ties (e.g., *now, I am going to talk about…, In this text, I am going to convince you…).* Raters counted each instance of a device in each of these categories. The inter-rater reliability was again high (≥0.90 across all measures, and for both tasks). This measure is reported as a number of occurrences per 100 words to give an index of tie density, independent of text length. In addition, we also report text length, counting the number of words written in the final text and removing incomplete or crossed words.

## Results

Observed means for reader- and text-based measures across test (pre-test, post-test) and condition (Direct Instruction, Modeling, and control) are shown in **Table 2** (individual -writing task) and **Table 3** (collaborative writing task).

**Table 2:** Effects of intervention on performance in the individual writing assessment task.

	Direct Instruction		Modeling		Control	
	Pre-test	Post-test	Pre-test	Post-test	Pre-test	Post-test
Word count	81.5 (32.4)	80.8 (25.6)	92.4 (32.7)	91.4 (26.5)	72.0 (35.8)	65.8 (23.1)
Structure	1.05 (0.23)	2.89 (1.18)	1.06 (0.34)	2.55 (1.13)	1.03 (0.17)	1.86 (1.15)
Coherence	1.11 (0.31)	2.63 (1.15)	1.18 (0.39)	2.41 (1.10)	1.08 (0.28)	1.69 (0.95)
Overall quality	1.45 (0.69)	3.45 (1.37)	1.38 (0.65)	3.38 (1.30)	1.22 (0.28)	2.08 (1.18)
Sophisticated coherence devices	0.64 (0.96)	3.71 (2.67)	0.42 (0.59)	2.41 (2.44)	0.57 (0.82)	1.83 (2.53)

*Mean scores with standard deviation in parentheses.*

**Table 3:** Effects of intervention on performance in the collaborative writing assessment task.

	Direct Instruction		Modeling		Control	
	Pre-test	Post-test	Pre-test	Post-test	Pre-test	Post-test
Word count	71.8 (20.6)	64.8 (22.1)	75.5 (26.2)	83.3 (19.8)	55.3 (21.1)	68.1 (16.2)
Structure	1.24 (0.44)	2.28 (0.96)	1.16 (0.38)	3.47 (0.84)	1.06 (0.24)	2.17 (0.92)
Coherence	1.33 (0.48)	3.33 (0.86)	1.53 (0.51)	3.47 (0.84)	1.22 (0.43)	2.33 (0.84)
Overall quality	2.14 (0.66)	4.76 (1.09)	2.26 (0.73)	5 (1.16)	1.94 (0.64)	3.06 (0.94)
Sophisticated coherence devices	0.52 (0.92)	6.27 (3.58)	0.23 (0.45)	3.04 (2.80)	0.58 (0.78)	2.37 (2.10)

*Mean scores with standard deviation in parentheses.*

To evaluate intervention effects we tested linear mixed effect models with random by-student and by-class intercepts, and with condition (Direct Instruction, Modeling, Control), time (pre-test, post-test), and their interaction as fixed factors. This approach achieves the same end as performing a mixed-effects ANOVA, but allows for the possibility that variance is not homogenous across measurement occasions, a state of affairs that is likely in the present and similar contexts (Quené and Van den Bergh, 2004, 2008). Evidence of an effect of intervention comes from the interaction between condition and time-of-task. Each model therefore evaluated three planned contrasts: the two-way interaction between task (pre-test vs. post-test) and condition (each of Direct vs. control, Modeling vs. control, and Direct vs. Modeling). Statistical significance of these effects was evaluated against a t distribution with degrees of freedom corrected for the dependencies in the observations. We also report Cohen’s *d* as an indication of effect size, calculated within-condition difference between pre-test and post-test.

### Relationships among Measures

Correlations among dependent variables can be found in **Table 4**. As might be expected, quality measures were correlated, but these correlations are sufficiently low to suggest good discriminant validity.

**Table 4:** Correlations among reader-based and text-based measures at pre-test.

	Individual task			Collaborative task		
	Coherence	Quality	Complex coherence devices	Coherence	Quality	Complex coherence devices
Structure	0.46	0.64	0.23	0.57	0.49	-0.008
Coherence		0.51	0.32		0.67	-0.07
Quality			0.36			0.19

### Equivalence of Writing Skills at Pre-test

We first determined whether there was evidence of differences among three experimental conditions at pre-test. One-way ANOVA indicated no statistically significant differences between conditions for any of structure, coherence, quality and the use of sophisticated coherence devices, either for the individual or collaborative tasks (*F* ≤ 1.9, *p* ≥ 0.20 for all analyses). There was some evidence of pre-test differences in the length of students’ texts [Individual: *F*(2.12) = 3.6, *p* = 0.03; Collaboratively: *F*(2.55) = 4.1, *p* = 0.02].

### Intervention Effects – Pre-test vs. Post-test

#### Individual Writing

Looking first at the effects of intervention on performance in the individual writing tasks, we found no effect of intervention on the length of the texts produced by students. There were, however, clear effects on reader-based quality measures, with evidence of a greater improvement in performance relative to control group in both the Direct Instruction and Modeling conditions [Direct Instruction: Structure, *t*(120) = 4.0, *p* < 0.001, *d* = 2.6; Coherence, *t*(120) = 3.9, *p* < 0.001, *d* = 2.1; Overall Quality, *t*(120) = 4.1, *p* < 0.001, *d* = 1.9. Modeling: Structure, *t*(120) = 2.8, *p* = 0.007, *d* = 2.0; Coherence, *t*(120) = 2.9, *p* = 0.005, *d* = 1.6; Overall Quality, *t*(120) = 4.1, *p* < 0.001, *d* = 2.0]. Comparing the effects of Direct Instruction and Modeling gave no statistically significant differences.

Students in the Direct Instruction condition showed an increase in the use of sophisticated coherence devices compared with the control group [Direct Instruction, *t*(120) = 3.2, *p* = 0.002, *d* = 1.69]. Note that although the effect size appears large here, there was also a substantial increase in the use of these devices in the Control condition. We did not find a statistically significant effect for the Modeling, relative to control, and again there was no evidence of a statistically significant difference between the effects of the Modeling and Direct Instruction.

#### Collaborative Writing

Effects of intervention on performance in the writing-in-pairs task showed statistically significant improvement on all variables apart from text length [Direct Instruction: Structure, *t*(58) = 3.3, *p* = 0.002, *d* = 1.5; Coherence, *t*(58) = 2.9, *p* = 0.005, *d* = 3.0; Overall Quality, *t*(58) = 4.6, *p* < 0.001, *d* = 3.0; Coherence markers, *t*(58) = 4.5, *p* < 0.001, *d* = 2.6. Modeling: Structure, *t*(58) = 4.2, *p* < 0.001, *d* = 3.8; Coherence, *t*(58) = 2.7, *p* = 0.010, *d* = 0.68; Overall Quality, *t*(120) = 4.8, *p* < 0.001, *d* = 2.1; Coherence markers, *t*(58) = 2.0, *p* = 0.05, *d* = 1.7]. Comparing the effects of Direct Instruction and Modeling gave no statistically significant differences for structure, coherence and quality. Regarding the use of complex coherence devices, a significant difference was found favoring direct instruction condition compared with modeling [*t*(58) = 2.9, *p* = 0.005].

#### Role Effects

It is possible that students’ role when practicing in pairs during instruction – whether they were Helper or Writer – affected the extent to which they benefited from intervention. We tested this hypothesis by adding role, and its interaction with other factors, to our model. This did not significantly improve model fit. We therefore did not find evidence that role moderated the intervention effects.

#### Differential Effects

It is also possible that students’ writing ability, as measured by scores on the pre-test task, could moderate effects of the intervention. For example, it could be that although there was no evidence that within the population as a whole Direct Instruction benefits students more that Modeling, weaker students benefit more from Direct Instruction and stronger students more from Modeling (or perhaps the reverse). With this aim we conducted moderator regression analyses using Hayes’ implementation of the Johnson–Neyman technique (Johnson and Neyman, 1936; Hayes, 2013). This analysis examined the effect of pre-test score, as a continuous predictor, on the effect of condition on post-test score. We found no evidence that effects of pre-test score on performance differed reliably across condition.

## Discussion

The main purpose of this study was to compare the benefits of teaching upper-primary children planning and drafting strategies by either expert modeling or direct instruction. The pattern of results obtained in both collaborative and individual writing tasks confirm that both components of strategy-focused writing instruction are effective. Both experimental conditions showed greater gains in the quality of their texts on reader- and text-based measures, relatively to a control group that received non strategy-focused but text analytic instruction. In the present study we found benefits of strategy instruction after only two intervention sessions. This is in line with Fidalgo et al. (2015) who also found large, immediate benefits of students observing and reflecting on an expert model after two sessions in three different groups.

Improvement in text quality was not simply due to students writing longer compositions. The number of words written in all conditions in the present study did not significantly differ before and after intervention. Some previous studies have found that strategy-focused interventions result in an increase in the number of words written by the students (for reviews, see Graham and Harris, 2003; Graham, 2006; Harris et al., 2009; but see Harris et al., 2012; Torrance et al., 2015). The fact that text quality improvements were not dependent on students writing more words suggests that intervention effects are not readily explained simply in terms of an increase in students’ motivation.

The main aim of this study was, however, to determine the relative effects of direct instruction and modeling – two instructional components that are typically combined in strategy-focused instruction. Our findings did not indicate any statistically reliable differences between the effects of these two components: modeling and direct instruction proved similarly effective in improving the quality of students’ texts. The instructional content covered by these two conditions were the same. In both conditions, students were exposed to planning and drafting writing strategies associated with identifying audience needs, setting goals, generating and organizing content, and so forth. However, while in direct instruction the strategies were made explicit through mnemonics, in the modeling condition students inferred writing strategies from the observation of a model. Therefore, students in the modeling condition used these strategies but did not label them at any time. This is, to our knowledge, the first study to directly compare these forms of instruction. Previous studies have found that direct instruction, in the absence of modeling, can be effective in developing writing skills (Sawyer et al., 1992) albeit in struggling writers rather than the full-range classes that were the focus of the present study. Fidalgo et al. (2015) found that modeling without direct instruction can be effective in developing writing skills in six graders’ typically developing students. Our finding confirms that, for typically developing writers, both approaches, when applied in isolation, are effective. Note, however, that it is possible that if modeling had not been separated from other critical activities such as evaluation or elaboration (Braaksma et al., 2001), students in this condition could have outperformed students in direct instruction condition. This is what Sonnenschein and Whitehurst (1984) showed in their study, in which preschool students in observation plus evaluation condition performed better than their peers in the only observation condition. These results are consistent with findings reported by Fidalgo et al. (2011, 2015), in which modeling including self-reflection showed to be sufficient to improve writing skills in normally achieving upper primary students. However, we explicitly decided to focus only on modeling, removing the reflection component, to avoid the possible interference of the whole-class reflection and to guarantee that we test what students learned from their own observations and not from the others’ reflections. Crucially, however, our results showed that, at least in the present context, even without direct instruction or any formal reflection by the students, they still learned as well from modeling as they did from direct instruction.

The collaborative and individual writing tasks showed similar patterns of results regarding text quality. Students in both experimental conditions improved their texts when writing collaboratively as well as when they wrote individually, which was not previously practized. The only difference found between the two tasks was related to the use of sophisticated coherence devices. In the individual task students in the direct instruction condition showed a larger increase compared to their peers in the control group. On the other hand, in the collaborative task both experimental conditions showed improvements on more sophisticated coherence devices compared to the control group and these were also significantly greater in the direct instruction compared to the modeling group. However, this specific text-based indicator did not have any impact on global text quality measures, which did not reflect any significant difference between collaborative or individual tasks. Research comparing collaborative and individual writing has found evidence of a positive effect of collaboration on task performance, which supports the use of collaborative writing tasks (Sutherland and Topping, 1999; Yarrow and Topping, 2001; Storch and Wigglesworth, 2007; Wigglesworth and Storch, 2009). These studies found that the quality of children’s collaborative writing was significantly higher than that of their individual writing. However, in the present study we did not find any difference between collaborative and individual task. It may be the case that the quality of the feedback given to the writers by the helpers was poor due to the complexity of the strategies taught, the duration of the intervention, the fact that only one component was taught in each condition and the short period of time devoted to practize collaborative writing. For example, for helpers in the modeling condition giving high-quality feedback might be especially complicated, given that they should remember the model process to guide their partner (*“I remember that the model first thought in the audience and then tried to find reasons to convince them”)* instead of recalling a mnemonic representing planning or drafting steps, as it was the case of helpers in direct instruction condition (*“Before R-reasons, we need to think in A-audience”*). Also, although the pair work was clearly established, previous research on pairs work has documented differences among the way in which learners participated in writing together (e.g., Schultz, 1997; Storch, 2001), which might have an effect on the quality of the final outcome. Therefore, future research is needed to explore the quality of the feedback provided and the kind of relations established between students. A detailed analysis of the pair transcripts recorded during the writing activity may provide interesting information about these issues.

Additionally, the analyses of the students’ role during emulative practice in the experimental conditions did not show significant results. Thus, students playing “writer” or “helper” roles in collaborative practice seemed to benefit equally in both intervention conditions. This suggests that engagement with the instructional content – whether delivered directly or by modeling – is similar either if the student responds by producing a text or by coaching another student.

The failure to find a difference in the efficacy of the Modeling and Direct Instruction approaches appeared to be true across the range of student ability. It was not the case that for weak students, or for strong students, one intervention proved more effective than the other. This result is not in line with previous studies, in which stronger students, not sampled by Sawyer et al. (1992), may particularly benefit from modeling (Groenendijk et al., 2013). One possible explanation for the lack of differences in the present study might be because we did not include the data of struggling writers in the analysis or, actually, there were not many abilities differences between students. Additionally, this was not helped by the floor effects and low variability in students’ initial writing achievement found at pre-test in our study. In subsequent studies, measures with larger range scales should be considered.

We want to quality our overall conclusion – that teaching writing strategies by modeling and by direct instruction are equally effective – in two ways.

First is possible that the positive effects of both interventions might have resulted just from an increase in student motivation. This is plausible but, as we noted above, we did not find reliable increases in the quantity of text produced by students at post-test, which would be the most likely effect of an increase in motivation. It did appear that the students produced better quality text because they had developed an understanding of text features and text production strategies that improved the quality of their written expression.

The failure to find a difference between the Modeling and Direct Instruction conditions might, however, also have a motivational explanation. It is possible, for example, that direct instruction was better at helping students to understand and remember the writing strategies but modeling was better at motivating them. Again this is plausible but, we believe, unlikely. Motivational features were quite well-controlled in across both conditions: both were delivered by the same instructor and we do not have any reason to believe that the content or delivery of either of the two interventions was intrinsically more motivating. Both conditions were novel and both included activities that, anecdotally, students enjoyed. In fact, both conditions included teaching aimed to promote students’ motivation, although there is no way of knowing whether or not these motivational components were equally effective. Again, if the two conditions different in their motivational effects then we would expect to find differences across conditions in the amount that students wrote, and this was not that case, we would expect to find differential.

Second our research does not rule out the possibility that the effects of modeling and direct instruction condition are temporary, or that one of the interventions had more persistent effects than the other.

Finally, in the present study we randomly allocated intact classes, rather than students, to conditions. Random allocation of children to condition is sometimes see as a gold standard. However we do not believe that this is the case for research of the kind that we report here. If you put a random collection of students together and then teach them as a group, and particularly if you then make them work collaboratively as we did in the present study, you risk both substantially disrupting students’ ability to learn and generating findings that do not generalize to the whole-class situation in which teachers will need to apply the intervention. Students placed in a new group will devote attention to making friends, getting comfortable with their new classmates and possibly classroom, and so forth rather than to intervention content That is, some of the whole-class effects that we get if you do not randomly allocate are effects that you actually what to be there. If you randomly allocate student to condition and then teach whole classes, you will still get class-level effects, but these are effects – differential performance across classes as a result of unpredictable new group dynamics – are likely to reduce the benefit they get from the intervention and the generalizability of our findings.

In summary, our findings suggest that, for typically developing upper primary students, both modeling and direct instruction are effective to improve writing skills and result in significantly better quality argumentative texts, even after a short instructional period.

## Ethics Statement

The present study involved students, in all conditions, engaging in normal classroom activities and collection on normal performance data (i.e., nothing that would not happen normally during a school day). Unlike systems in, for example, the US, Spanish national guidelines and guidelines at University of León where the research was based, do not require that research of this kind go before an ethics panel. They require that the researcher commits to conduct research under the Code of Ethics of the World Medical Association (Declaration of Helsinki) (Williams, 2008). Plans for the present research were scrutinized and accepted both by the Spanish national research committee of Educational Sciences Area and the University of León Vice-Rector for research. They were discussed in detail with, and approved by, the schools in which the research was conducted. Additionally, parents were informed of research aims via letters in which they gave written informed consent. They were given the opportunity to express concerns and to request that their children’s data not be included in the study.

## Author Contributions

All authors listed have made a substantial, direct and intellectual contribution to the design of the work, analysis and data interpretation, drafting and revising it critically and approved it for publication.

## Conflict of Interest Statement

The authors declare that the research was conducted in the absence of any commercial or financial relationships that could be construed as a potential conflict of interest.
